# Screening for Oropouche virus in wild bird samples collected from wildlife rehabilitation centers, Florida, United States, 2021–2024

**DOI:** 10.3389/fpubh.2026.1799390

**Published:** 2026-04-23

**Authors:** Brent C. Newman, Rebecca H. Hardman

**Affiliations:** 1 Division of Vector-Borne Diseases, Centers for Disease Control and Prevention, Fort Collins, CO, United States; 2 Fish and Wildlife Research Institute, Florida Fish and Wildlife Conservation Commission, St. Petersburg, FL, United States

**Keywords:** arbovirus, *Culicoides paraensis*, emerging disease, Oropouche virus, wildlife

## Abstract

Oropouche virus (OROV) is an emerging orthobunyavirus whose range has recently expanded in the Americas, with recent outbreaks in Cuba and travel-associated cases reported in the United States, raising concerns about the potential for its establishment in the United States. While the primary vector, *Culicoides paraensis* (Diptera: Ceratopogonidae), is distributed throughout the southern United States, the role of avian hosts in the dispersal and maintenance of OROV is unknown. To address this knowledge gap, we conducted molecular screening for OROV in North American birds. We screened tissue samples from 81 individual birds representing 23 species opportunistically collected across Florida during 2021–2024. Sampling included migrants with ranges extending into regions with documented OROV transmission, partial migrants, and synanthropic resident species capable of influencing local transmission. All samples tested negative for OROV by RT-qPCR though such results do not eliminate the potential for OROV infection in these bird species. Our testing establishes a baseline for monitoring the northward expansion of this arbovirus at the human-wildlife interface in North America.

## 1 Introduction

Oropouche virus (OROV), an orthobunyavirus in the family Peribunyaviridae (Simbu serogroup), was first identified in Trinidad and Tobago in 1955 and is endemic to the Amazon basin region of South America (1, 2). Although OROV typically causes self-limiting febrile illness (Oropouche fever, Oropouche virus disease) characterized by severe headaches, myalgia, arthralgia, retro-orbital pain and in some cases, photophobia, nausea/vomiting, chills, and maculopapular rashes, it has been responsible for multiple epidemics and an estimated 500,000 cases in Central and South America since its discovery (3, 4). Oropouche fever typically lasts 2–7 days with symptoms recurring in up to 60% of patients (5, 6). Vertical transmission of OROV in humans has occurred, which has resulted in fetal deaths and congenital abnormalities including microcephaly (6, 7). Beginning in late 2023, OROV exhibited a significant range expansion beyond the Amazon basin to non-endemic regions of Central and South America, with an outbreak of human disease cases reported for the first time from Cuba during 2024 (5–7). The reemergence and northward expansion of OROV in the Americas has been attributed to a variety of factors, primarily anthropogenic land use change and a novel reassortant strain (AM0088) associated with enhanced viral fitness and immune evasion (5, 8). In the United States, over 100 travel-associated cases of OROV have been reported, primarily from travelers returning to Florida from Cuba, raising concerns about the potential for introduction and local establishment of this emergent arboviral disease (6).

Research indicates that OROV transmission ecology is maintained through a sylvatic cycle involving sloths and non-human primates, and an urban cycle, primarily driven by the biting midge *Culicoides paraensis* (Diptera: Ceratopogonidae) (9, 10). Within these cycles, the role of avian hosts remains uncertain, although evidence of OROV exposure [hemagglutination inhibition (HI) antibodies] has been detected in both domestic and wild birds across a variety of land use gradients throughout the Amazon Basin, which indicates that birds might serve as amplifying hosts or possibly dispersal mechanisms (11, 12).

The risk of autochthonous transmission in the United States is driven by the potential convergence of all three components of the disease triad: (1) presence of the primary vector, *C. paraensis*, whose known range encompasses 24 states and overlaps with all major migratory bird flyways in North America (9, 13) (2) avian hosts that might serve as amplifying or dispersal hosts; and (3) the OROV pathogen. Migratory bird-mediated dispersal of arboviruses-a mechanism demonstrated for West Nile virus (WNV) (14, 15)-could similarly facilitate OROV introduction and establishment in the United States. To address the need for baseline screening, we tested tissue samples from 23 avian species, both resident and migratory, collected across Florida during 2021–2024. Our primary objectives were to (1) detect active viral infection in sentinel wildlife populations, and (2) assess the potential for birds to introduce and influence OROV transmission cycles in the United States.

## 2 Materials and methods

### 2.1 Sample collection

**Figure 1:**
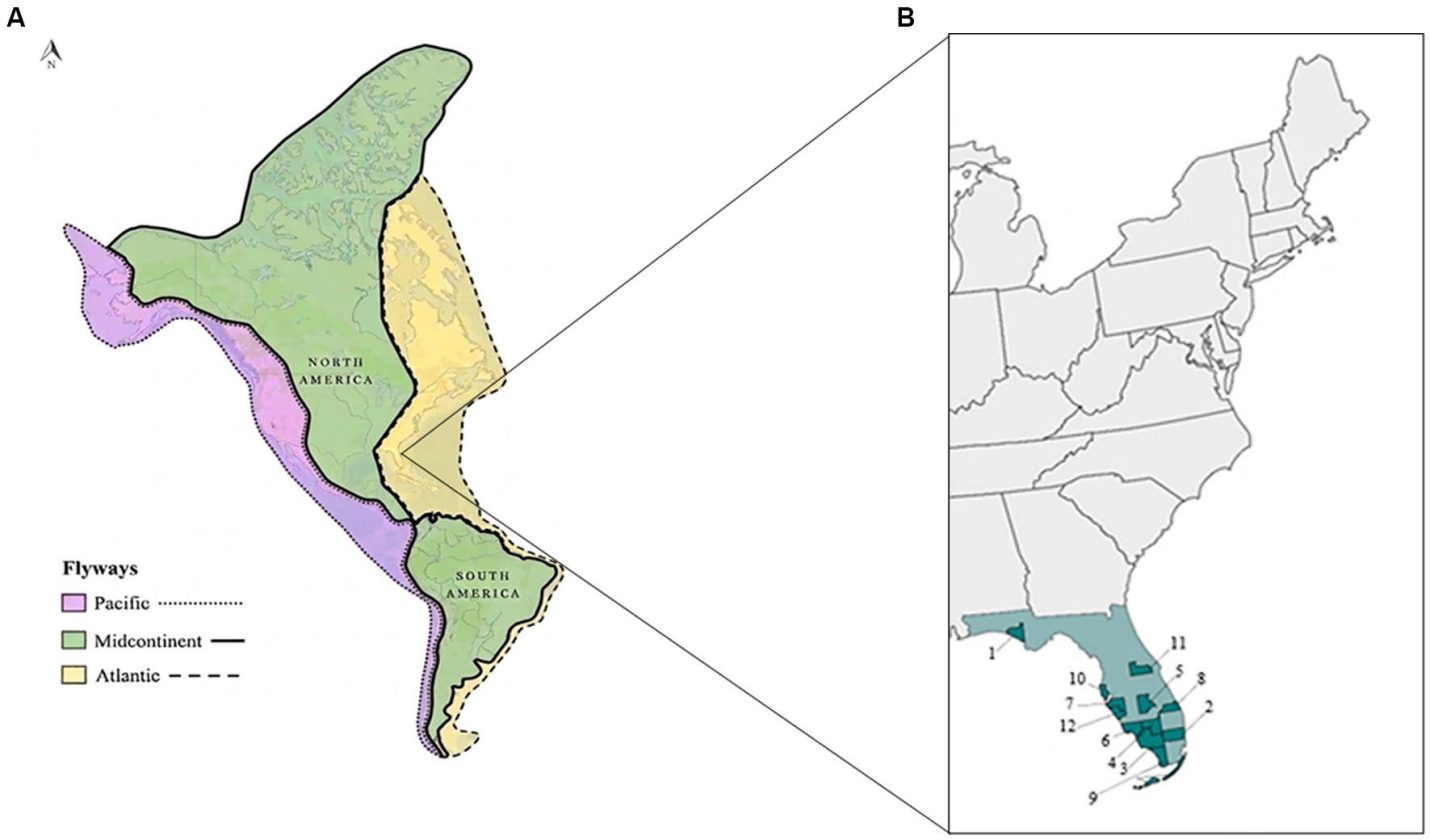
**(A)** Major migratory bird flyways across the Americas highlighting the Atlantic Flyway (yellow with dashed border) where sampling was conducted (adapted from: Shorebird Americas Flyway boundaries, U.S. Fish & Wildlife Service, Public Domain, https://gis-fws.opendata.arcgis.com/maps/6e12477fea9c44d3bc34e20f6216b58e/about). **(B)** Sample collection locations for Oropouche virus screening in wild birds in Florida (green shading) with counties numbered [Bay (1), Broward (2), Collier (3), Hendry (4), Highlands (5), Lee (6), Manatee (7), Martin (8), Monroe (9), Pinellas (10), Orange (11), and Sarasota (12)].

Sampling was conducted in Florida to identify presence of OROV RNA in avian hosts and assess potential for active infection across the Atlantic migratory flyway (Figure 1A). Avian tissue samples (kidney, *n* = 35; liver, *n* = 32; spleen, *n* = 31; *N* = 98) were opportunistically collected from 81 individual birds, representing 23 species, euthanized at wildlife rehabilitation centers because of welfare reasons (e.g., injury/inability to be rehabilitated) as determined by animal care staff during October 2021–July 2024. Following necropsy, tissue samples were stored at −80 °C and shipped on dry ice for RT-qPCR analysis. Samples were collected across 12 counties: Bay (*n* = 1), Broward (*n* = 1), Collier (*n* = 37), Hendry (*n* = 2), Highlands (*n* = 1), Lee (*n* = 13), Manatee (*n* = 2), Martin (*n* = 1), Monroe (*n* = 7), Pinellas (*n* = 2), Orange (*n* = 3), and Sarasota (*n* = 11) (Figure 1B). Florida specimens included raptors and owls [Broad-winged Hawk (*Buteo platypterus*; *n* = 1), Cooper’s Hawk (*Astur cooperii*; *n* = 2), Red-shouldered Hawk (*Buteo lineatus*; *n* = 3), Sharp-shinned Hawk (*Accipiter striatus*; *n* = 1), Barn Owl (*Tyto futcata*; *n* = 1), Burrowing Owl (*Athene cunicularia*; *n* = 35), Eastern Screech-Owl (*Megascops asio*; *n* = 1), Great-horned Owl (*Bubo virginianus*; *n* = 1), Osprey (*Pandion haliaetus*; *n* = 1)], seabirds [Anhinga (*Anhinga anhinga*; *n* = 1), Black Skimmer (*Rynchops niger*; *n* = 5), Brown Pelican (*Pelecanus occidentalis*; *n* = 3), American Oystercatcher (*Haematopus palliates*; *n* = 1), Muscovy Duck (*Cairina moschata*; *n* = 1), Common Tern (*Sterna hirundo*; *n* = 2), Royal Tern (*Thalasseus maximus*; *n* = 4), Sandwich Tern (*Thalasseus sandvicensis*; *n* = 6), Laughing Gull (*Leucophaeus atricilla*; *n* = 6), Lesser Black-backed Gull (*Larus fuscus*; *n* = 1)], wading birds [White Ibis (*Eudocimus albus*; *n* = 1)], and other native species [Sandhill Crane (*Antigone canadensis*; *n* = 1), Fish Crow (*Corvus ossifragus*; *n* = 2), and Boat-tailed Grackle (*Quiscalus major*; *n* = 1)] (Table 1). Sample collection was conducted by the Florida Fish and Wildlife Conservation Commission State Wildlife Veterinarian under institutional authority and oversight as part of ongoing wildlife health and mortality surveillance activities. While the majority of samples (2021–2022) were collected prior to the documented re-emergence and range expansion of OROV human cases in 2023, screening of these banked specimens was initiated as part of proactive OROV emergency response efforts.

**Table 1:** Summary of avian samples screened for Oropouche virus RNA in Florida, 2021–2024, arranged by bird species, collection location (county) and years, migration status (R, resident; M, migratory; P, partial migration), total count, and sample types screened (KDY, kidney; LVR, liver; SPLN, spleen).

Species	Common name	Florida county	Years	Migration status^a^	Count	KDY	LVR	SPLN
*Haematopus palliatus*	American Oystercatcher	Sarasota	2021	P	1	1	1	—
*Tyto furcata*	American Barn Owl	Martin	2023	R	1	—	—	1
*Anhinga anhinga*	Anhinga	Pinellas	2023	R	1	—	—	1
*Rynchops niger*	Black Skimmer	Collier, Lee, Orange, Pinellas	2023–24	R	5	2	2	1
*Quiscalus major*	Boat-tailed Grackle	Highlands	2023	R	1	1	—	—
*Buteo platypterus*	Broad-winged Hawk	Monroe	2024	M	1	1	1	—
*Pelecanus occidentalis*	Brown Pelican	Bay, Monroe	2023–24	R	3	2	—	2
*Athene cunicularia*	Burrowing Owl	Collier, Hendry, Lee	2023–24	R	35	15	22	7
*Sterna hirundo*	Common Tern	Collier	2024	R	2	—	—	2
*Astur cooperii*	Cooper’s Hawk	Collier, Manatee	2023	P	2	2	1	—
*Megascops asio*	Eastern Screech-Owl	Collier	2024	R	1	1	—	—
*Corvus ossifragus*	Fish Crow	Collier	2024	R	2	1	1	1
*Bubo virginianus*	Great Horned Owl	Hendry	2024	R	1	—	—	1
*Leucophaeus atricilla*	Laughing Gull	Collier, Sarasota	2024	R	6	—	—	6
*Larus fuscus*	Lesser Black-backed Gull	Collier	2024	P	1	—	—	1
*Cairina moschata*	Muscovy Duck	Broward	2021	R	1	1	—	—
*Pandion haliaetus*	Osprey	Sarasota	2023	R	1	—	1	—
*Buteo lineatus*	Red-shouldered Hawk	Manatee, Sarasota	2024	R	3	3	2	2
*Thalasseus maximus*	Royal Tern	Collier, Lee, Monroe	2024	R	4	3	—	1
*Antigone canadensis*	Sandhill Crane	Orange	2023	P	1	1	—	—
*Thalasseus sandvicensis*	Sandwich Tern	Collier	2024	P	6	—	—	6
*Accipiter striatus*	Sharp-shinned Hawk	Collier	2023	M	1	1	—	—
*Eudocimus albus*	White Ibis	Orange	2024	R	1	—	1	—
Total					81	35	32	32

a Migration status determined using the Birds of the World website (www.birdsoftheworld.org), ^©^Cornell Lab of Ornithology.

Visualizations were generated in R (v4.4.1) using tidyverse, sf, tigris, and ggplot2 packages (16–20).

### 2.2 Molecular screening

RNA extractions from tissue samples were performed using the QIAamp Viral RNA Mini Kit following the manufacturer’s protocol. Tissue samples (~5.0–20.0 mg) were homogenized before extraction using beads (Tungsten Carbide Beads, 3 mm, QIAGEN) in a QIAGEN TissueLyser III. RT-qPCR was performed on a CFX96 Real-Time PCR Detection System (Bio-Rad Laboratories, Hercules, CA) using the QuantiTect Probe RT-PCR Kit and targeting the OROV S segment with primers OROV_FNF (5′-TCCGGAGGCAGCATATGTG-3′) and OROV_FNR (5′-ACAACACCAGCATTGAGCACTT-3′), and probe OROV_FNP (5’-FAM-CATTTGAAGCTAGATACGG-NFQ-MGB-3′) (21). Positive controls included OROV isolates 240023 (isolate from traveler returning to the United States from Cuba in 2024) and TRVL 9760 (original 1955 Trinidad strain) obtained from the Centers for Disease Control and Prevention Arbovirus Reference Collection. Samples were considered positive at a cycle threshold (Ct) ≤37.

## 3 Results

All avian tissue samples (*n* = 98) tested negative for OROV RNA by RT-qPCR (Table 1). Both positive controls were included on both testing plates and performed as expected. Isolate 240023 Ct values were 29.12, 30.24, 30.66 (plate 1) and 29.05, 30.34, 31.74 (plate 2) for dilutions 10^−1^ through 10^−3^, respectively. Isolate TRVL 9760 Ct values were 27.24, 29.42, 30.37 (plate 1) and 27.40, 29.15, 30.37 (plate 2) for dilutions 10^−1^ through 10^−3^, respectively. Of the 23 different species screened, nine of these are considered migratory or partial migrants (species in which part of the population remains resident and the remainder migrates based on environmental conditions, resource availability, etc.) with ranges extending to the Caribbean—including Cuba—and South America (22). The remaining 14 species are considered resident and occur across rural to urban gradients (22).

## 4 Discussion

This study is the first molecular screening effort to detect active OROV infection in avian populations in North America. The absence of detectable viral RNA in the 81 screened birds establishes a baseline for monitoring the northward expansion of OROV, although it does not preclude the role of these species, or more broadly, avian host, in the range expansion of this emergent pathogen. Despite our results, which reflect absence of detectable infection rather than species susceptibility, the ecological mechanisms for the introduction and establishment of OROV in North America remain plausible based on the convergence of host, vector, and pathogen at the human-wildlife interface.

At least eight avian species screened for our study are migratory or partial migrants with ranges extending to the Caribbean, Central America, and South America-regions where OROV is endemic or has recently established (9, 22). These movements represent potential routes of entry, mirroring dispersal pathways documented for other arboviruses previously introduced to the United States, such as WNV (14, 23). However, introduction of the pathogen alone is insufficient for establishing a self-sustaining transmission cycle in North American wildlife. Such establishment requires the convergence of susceptible avian (wildlife) hosts, competent vectors, and environmental conditions that enable vector-host contact in both space and time.

Local establishment risks also exist and are compounded by resident synanthropic species (e.g., Boat-tailed Grackles, Fish Crows, Muscovy Ducks), which might influence transmission cycles via amplification or maintenance of OROV in wildlife and vector populations. In arboviral systems, host importance is often determined by ecological abundance and vector contact rates rather than physiological competence alone (24). *Culicoides paraensis* exhibits opportunistic feeding patterns on avian and mammalian hosts with documented anthropophagic behavior in urban areas (25, 26). Additionally, other North American *Culicoides* species exhibit a primary preference for avian hosts including *C. arboricola*, *C. haematopotus*, and *C. crepuscularis* (27–29). However, to our knowledge, the vector competence of these species for OROV has not been evaluated. Synanthropic birds could therefore influence transmission even if their viremia levels differ from primary sylvatic hosts, particularly given the documented avian host preferences of multiple North American *Culicoides* species.

### 4.1 Historical evidence of avian OROV exposure

**Figure 2:**
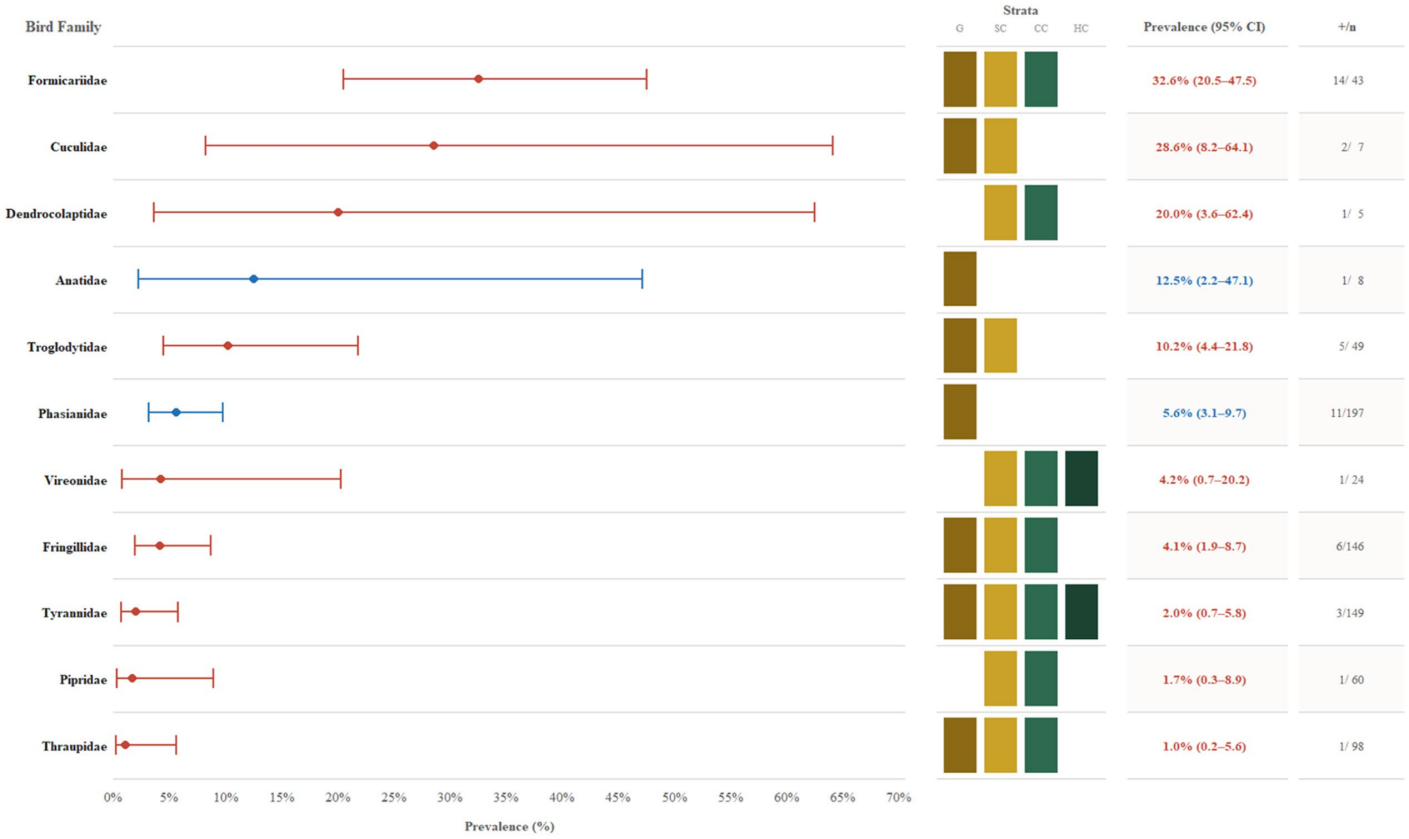
Forest plot of Oropouche virus (OROV) seroprevalence by avian family using data from Pinheiro et al. (11). Point estimates represent the percentage of seropositive individuals within bird families, ordered by highest to lowest prevalence, with error bars indicating 95% Wilson score confidence intervals. Red markers and text denote wild bird families, while blue denotes domestic families (Phasianidae, Anatidae). The central grid highlights the vertical ecological strata occupied by each family: G (Ground), SC (sub-canopy; 1–5 m), CC (central canopy/mid-story; 5–15 m), and HC (high canopy; >15 m). Adjacent columns display the prevalence estimates (with 95% CI) and the count of positive individuals by the total number tested (*n*).

While our molecular screening detected no active OROV infection, historical serological studies provide important background for understanding the potential for avian involvement in OROV natural transmission cycles. A survey of 886 birds comprising 21 families was conducted in Mojuí dos Campos, Pará, Brazil, during an outbreak investigation (11). This study demonstrated varying degrees of OROV exposure, including HI antibodies in domestic birds (*n* = 205) such as Phasianidae (domestic chickens) at ~5.6% (95% CI, 3.1–9.7; 11/197) (11). Among wild bird families sampled (*n* = 681), the highest OROV seroprevalence rates were documented in Formicariidae (ant-thrushes) at 32.6% (95% CI, 20.5–47.5; 14/43), followed by Cuculidae (cuckoos) at 28.6% (95% CI, 8.2–64.1; 2/7), and Dendrocolaptidae (woodcreepers) at 20.0% (95% CI, 3.6–62.4; 1/5) (Figure 2). These findings indicate that avian families occupying lower-level forest strata (~0–10 m) might be exposed or contribute disproportionately to OROV transmission cycles during epidemic situations.

**Figure 3:**
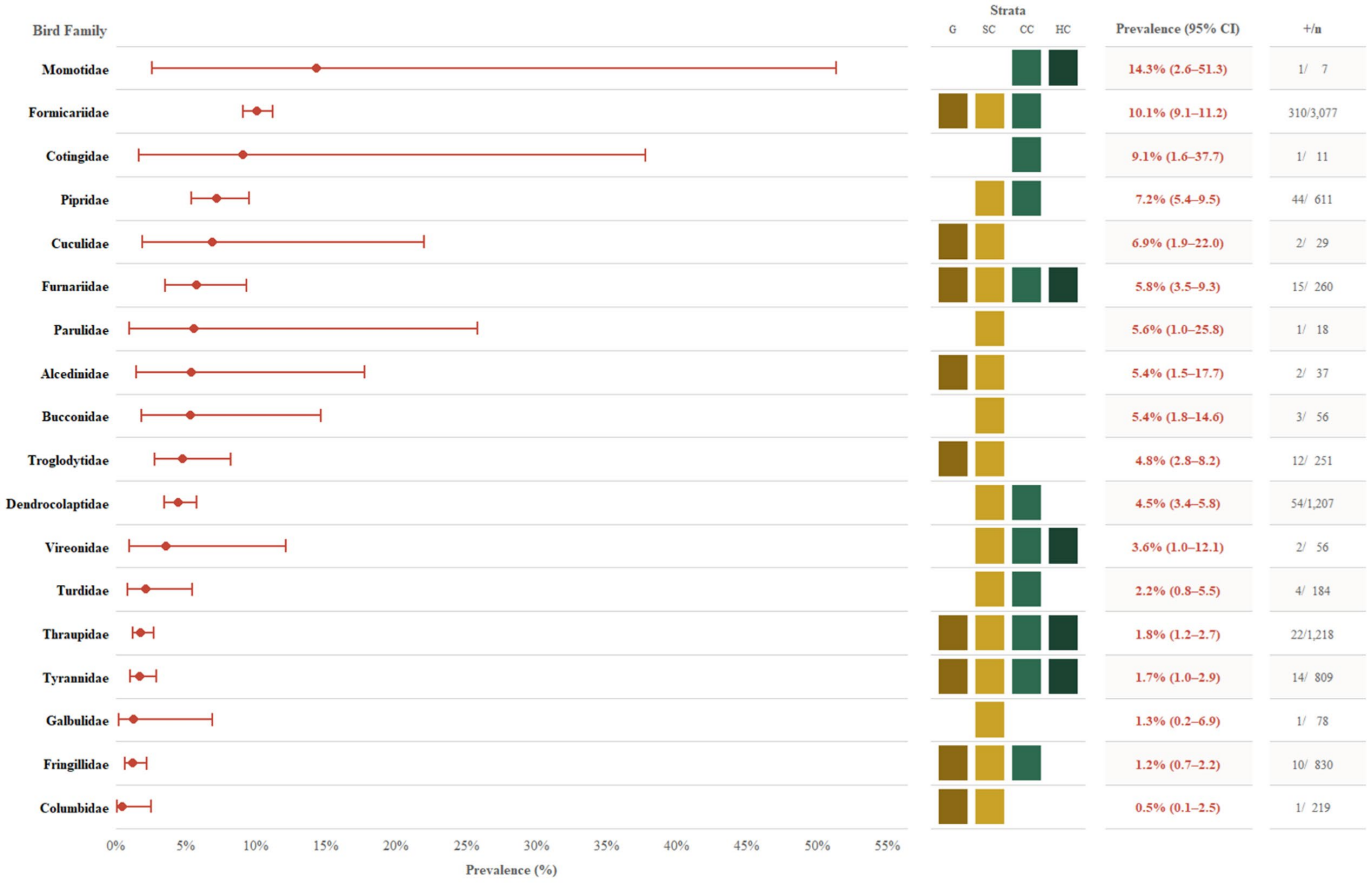
Forest plot of Oropouche virus (OROV) seroprevalence by avian family using data from Dégallier et al. (12). Point estimates represent the percentage of seropositive individuals within bird families, ordered by highest to lowest prevalence, with error bars indicating 95% Wilson score confidence intervals. All markers denote wild bird families (*n* = 8,958 total specimens). The central grid highlights the vertical ecological strata occupied by each family: G (ground), SC (sub-canopy; 1–5 m), CC (central canopy/mid-story; 5–15 m), and HC (high canopy; >15 m). Adjacent columns display the prevalence estimates (with 95% CI) and the count of positive individuals by the total number tested (*n*).

This pattern of disproportionate exposure in birds utilizing low-strata environments was not limited to outbreak conditions. A comprehensive serological survey of 40 wild bird families (*N* = 12,423 samples) conducted between 1964 and 1989 across the Amazon Basin region of Brazil found a total OROV seroprevalence of 3.86%, with some of the highest levels of exposure (HI antibodies) again, identified in Formicariidae (10.1%; 95% CI, 9.1–11.2; 310/3,077) along with other bird families that primarily utilize ground to low level forest strata (12) (Figure 3). Additionally, live virus was isolated from a single Ruddy Ground Dove (*Columbina talpacoti*) during this study, with species-level analysis demonstrating that understory/ground-foraging specialists, such as the East Amazonian Fire-eye (*Pyriglena leuconota*) and Rufous-throated Antbird (*Gymnopithys rufigula*), exhibited some of the highest OROV seroprevalence rates at 30.5% (68/223) and 23.7% (37/156), respectively (12). These findings demonstrate that vector-host contact and OROV transmission risk likely converge at or near ground-level microhabitats. The historical serological evidence, representing past exposure rather than active infection, provides ecological context that supports opportunistic screening of North American birds as outbreaks of OROV are documented in new areas.

### 4.2 Current North American context

Transmission risk at the avian-human interface is further defined by vector presence and host plasticity. Sampled counties in Florida are located within the Atlantic Flyway, which creates a focal point for resident and migratory population interfaces (30). Additionally, the primary urban vector of OROV, *C. paraensis*, has been documented from Hendry, Highlands, and Orange counties, Florida, which directly overlapped our sampling (13). Although *C. paraensis* drives epidemic transmission in endemic areas, the role of secondary North American vectors in OROV transmission remains unclear. *Culex quinquefasciatus* (Diptera: Culicidae) demonstrates low biological competence in laboratory settings, with detection of OROV in wild-caught specimens during outbreaks in Brazil indicating natural infection capability (31–33). However, the epidemiological significance of *C. quinquefasciatus* in OROV transmission dynamics requires further investigation. Similarly, other biting midges native to North America, such as *C. sonorensis,* are highly competent OROV vectors but have strong mammalophilic feeding preferences, potentially limiting their role in avian-mediated transmission cycles. Therefore, more systematic surveillance across different geographic regions and vector-host transmission cycles are needed to fully evaluate OROV introduction and spillover risk in North America (34).

### 4.3 Limitations

While more comprehensive molecular and serological surveys across expanded geographic regions and longer temporal scales would provide a more robust assessment of avian OROV exposure in North America, the rapid geographic expansion of OROV cases outside its endemic range, particularly recent outbreaks in Cuba, necessitated timely sample collection. Our study leveraged partnerships with a state wildlife agency and rehabilitation centers to screen migratory and resident bird species from areas near/within the distribution of *C. paraensis* in the United States, and in proximity to outbreak regions (Florida-Cuba). This approach enabled rapid response to an emerging arboviral threat.

## 5 Conclusion

This study establishes a molecular baseline for OROV infection in North American wildlife. Although no active infection was detected in any samples, this absence of detection does not eliminate the potential for avian involvement in OROV transmission. Historical evidence of avian exposure in endemic regions (Brazil) (11, 12) indicates that continued monitoring of birds may be a valuable tool for informing future preparedness. As OROV continues to undergo genomic reassortment and geographic expansion into non-endemic regions (31), proactive monitoring strategies that integrate sentinel wildlife screening and targeted vector surveillance at high-risk interfaces are essential to detect potential introduction events and protect public health.

## Data Availability

The original contributions presented in the study are included in the article/supplementary material, further inquiries can be directed to the corresponding author.
